# Local changes in neocortical circuit dynamics coincide with the spread of seizures to thalamus in a model of epilepsy

**DOI:** 10.3389/fncir.2014.00101

**Published:** 2014-09-03

**Authors:** Florian B. Neubauer, Audrey Sederberg, Jason N. MacLean

**Affiliations:** ^1^Department of Neurobiology, The University of ChicagoChicago, IL, USA; ^2^Department of Physiology, University of BernBern, Switzerland; ^3^Committee on Computational Neuroscience, The University of ChicagoChicago, IL, USA

**Keywords:** epilepsy model, seizure generalization, neocortex, thalamus, patch-clamp, two-photon imaging, mouse

## Abstract

During the generalization of epileptic seizures, pathological activity in one brain area recruits distant brain structures into joint synchronous discharges. However, it remains unknown whether specific changes in local circuit activity are related to the aberrant recruitment of anatomically distant structures into epileptiform discharges. Further, it is not known whether aberrant areas recruit or entrain healthy ones into pathological activity. Here we study the dynamics of local circuit activity during the spread of epileptiform discharges in the zero-magnesium *in vitro* model of epilepsy. We employ high-speed multi-photon imaging in combination with dual whole-cell recordings in acute thalamocortical (TC) slices of the juvenile mouse to characterize the generalization of epileptic activity between neocortex and thalamus. We find that, although both structures are exposed to zero-magnesium, the initial onset of focal epileptiform discharge occurs in cortex. This suggests that local recurrent connectivity that is particularly prevalent in cortex is important for the initiation of seizure activity. Subsequent recruitment of thalamus into joint, generalized discharges is coincident with an increase in the coherence of local cortical circuit activity that itself does not depend on thalamus. Finally, the intensity of population discharges is positively correlated between both brain areas. This suggests that during and after seizure generalization not only the timing but also the amplitude of epileptiform discharges in thalamus is entrained by cortex. Together these results suggest a central role of neocortical activity for the onset and the structure of pathological recruitment of thalamus into joint synchronous epileptiform discharges.

## Introduction

During the onset of generalized epileptic seizures, an initial focus of aberrant activity arises in a spatially restricted local network of brain cells and subsequently recruits distributed, hitherto unaffected brain areas into joint epileptic discharges (Berg et al., 2010). Seizure generalization remains poorly understood partly because of the complexity of neocortical circuit dynamics that arise during epileptic discharges and the multitude of molecular and cellular alterations that can contribute to aberrant brain activity (Prince, 1983; Wong and Prince, 1990; Steriade et al., 1993; Pinto et al., 2005; Trevelyan et al., 2007). A better understanding of the focal causation of generalized seizures due to the dynamic interplay of short connections, i.e., local circuits, and long-range connectivity could help to explain why normal information processing of the brain becomes impaired during seizure generalization and could be of direct benefit for clinical application. For example, seizure prediction (Mormann et al., 2007; Lehnertz, 2008; Carney et al., 2011) and therapeutic brain stimulation approaches (Fisher et al., 2010; Fisher, 2011; Morrell, 2011) potentially could be improved based on a better understanding of the interplay between local and distal connectivity of the brain during the spread of seizures.

*In vitro* studies of the neurobiological correlate of seizure generalization require an epilepsy model which comprises anatomically distinct but recurrently connected brain areas and a uniformly induced imbalance between excitation and inhibition in order to allow for multiple distributed initiation sites similar to those found in generalized epilepsies (Berg et al., 2010). The combination of the acute thalamocortical (TC) brain slice preparation with the zero-Mg^2+^ induction model of epileptiform discharges meets both requirements. The TC slice preserves reciprocal connectivity between thalamus and cortex (Agmon and Connors, 1991; Theyel et al., 2010). Further, the TC loop is known to be predisposed by its normal function to synchronized discharges during epileptic seizures (Steriade and Amzica, 1994; Chang and Lowenstein, 2003; Huguenard and McCormick, 2007; Beenhakker and Huguenard, 2009). Initiation of epileptiform activity in the zero-Mg^2+^ model of epilepsy has been associated with enhanced excitation due to the removal of the magnesium block at NMDA receptors (Walther et al., 1986; Mody et al., 1987; Traub et al., 1994) and has been shown to be partly caused by decreased inhibition (Whittington et al., 1995; Trevelyan et al., 2007). Using this model, all cells contained in an *in vitro* preparation are subjected to the same ionic condition of hyperexcitability and therefore focal sites of seizure generation are distributed within areas of comparable connectivity such as different cortical columns (Wong and Prince, 1990). The TC slice in combination with the zero-Mg^2+^ paradigm can be used to study the interplay of anatomically distant but functionally connected brain areas during and after seizure generalization (Coulter and Lee, 1993).

Here we evaluate both thalamic and cortical local circuit activity during epileptiform discharges in the zero-Mg^2+^ *in vitro* model of epilepsy. We employ high-speed multi-photon calcium imaging of up to 1300 neurons (Sadovsky et al., 2011) to capture the dynamics of local neuronal circuits in thalamus or cortex with single-cell resolution. Using simultaneous dual patch-clamp recordings we assess long-range seizure spread between the two brain areas. We find that initial seizure onset is localized to neocortex, consistent with previous reports (Steriade and Contreras, 1998; Meeren et al., 2002; Polack et al., 2007). Pathophysiological recruitment of thalamus into synchronized epileptiform discharges by cortex coincides with increased correlation of activity within the local neocortical circuit of neurons. This increase in intracortical correlation is independent of the presence of thalamus. After the recruitment of thalamus by cortex into synchronous epileptiform discharges has stabilized, thalamus follows cortex in a functionally coupled manner, typified by a strong correlation between discharge amplitudes in both brain structures. Our results indicate that the generalization of epileptic discharges in the brain occurs simultaneously with a functional change in the area of primary, focal seizure onset and that the intensity of aberrant activity in primary and secondary areas remains correlated after epileptic activity has generalized.

## Materials and methods

### Slice preparation and calcium-sensitive dye staining

Four-hundred-fifty μm thick TC slices were prepared with a vibratome (VT1000S, Leica) from postnatal day 13 to 16 C57BL/6 mice. This preparation preserves both intact TC (Agmon and Connors, 1991) and corticothalamic (Theyel et al., 2010) connectivity. Control slices without TC connectivity were prepared in the same way except that thalamus was carefully cut away with a bent needle. All procedures were approved by the Institutional Animal Care and Use Committee at the University of Chicago. Mice were anesthetized by intraperitoneal injection of ketamine-xylazine. Brains were cut in ice-cold modified ACSF that contained (in mM) 205 sucrose, 3 KCl, 26 NaHCO_3_, 1 NaH_2_PO_4_, 0.5 CaCl_2_, 6 MgSO_4_, 25 dextrose. Subsequently, slices were incubated for 30–40 min at 35°C in a solution containing (in mM) 123 NaCl, 3 KCl, 26 NaHCO_3_, 1 NaH_2_PO_4_, 1 CaCl_2_, 3 MgSO_4_ and 25 dextrose. All solutions were equilibrated with 95% O_2_/5% CO_2_. For visualization of action potential generation slices were bulk loaded with the calcium-sensitive dye Fura-2AM. For cortical imaging this was done as previously described (MacLean et al., 2005). For thalamic imaging a modified staining technique was used to account for the higher density of thalamic tissue; a pH-stabilized dye solution prepared by dissolving 50 μg Fura-2AM in 6.7 μl of 20% (w/v) Pluronic F-127 in DMSO and adding 127 μl of a HEPES buffered solution (in mM: 125 NaCl, 2.5 KCl, 10 HEPES, adjusted with NaOH to pH 7.25), was manually injected into thalamus under visual control (10x objective) using a back-filled glass micropipette with an opening diameter of 18–22 μm connected via air-filled tubing to a 50 ml syringe. This staining variant was done at RT in standard ASCF containing (in mM) 123 NaCl, 3 KCl, 26 NaHCO_3_, 1 NaH_2_PO_4_, 2 CaCl_2_, 2 MgSO_4_, 25 dextrose, followed by a wash-out period of extracellular dye of >1 h.

### Induction of epileptiform activity

Epileptiform activity was induced using slice superfusion with zero-Mg^2+^-ACSF, which was prepared omitting MgSO_4_ from the standard ACSF recipe. Experiments were conducted at RT (19–24°C).

### Electrophysiology

Whole-cell current-clamp recordings in cortex and/or thalamus were carried out using Multiclamp 700 B amplifiers (Molecular Devices). Patch pipettes 5–8 MΩ were filled with (in mM) 135 K-gluconate, 4 MgCl_2_, 10 HEPES, 2 Na_2_-ATP, 0.3 Na-GTP, 10 Na_2_-phosphocreatine and 0.5% (w/v) biocytin, adjusted with KOH to pH 7.25. Custom written software (LabVIEW) was used for controlling Multiclamp and stimulation electrodes via a DAQ board (6733; National Instruments). For each TC cell the presence of rebound spiking following hyperpolarizing current pulses was confirmed. Thalamic extracellular stimulation was delivered as a train of 4 pulses (0.2 ms) at 40 Hz by a bipolar iridium/platinum electrode (FHC, #CE2C55) inserted at the medial border of the ventral posteromedial nucleus.

### High-speed two-photon imaging

Two-minute long two-photon imaging recordings providing activity profiles for up to 1300 cells were acquired at intervals of 5–10 min, starting after electrophysiological recordings were established. Imaging data was acquired with a custom-built multi-photon microscope, using a femtosecond pulsed Chameleon Ultra II Ti:sapphire laser (Coherent, *λ* = 790 nm). Galvomotors were given voltage commands at 312.5 kHz via a DAQ board (6733; National Instruments) and custom software (LabVIEW). To maximize the speed of fluorescence measures from large populations of neurons using a standard two-photon microscope we employed the Heuristically Optimal Path Scanning method (Sadovsky et al., 2011) which largely eliminates the off-target laser travel of conventional raster scans.

### Analysis

#### Detection of epileptiform discharges

Electrophysiological and imaging raw time series were analyzed off-line with custom MATLAB scripts. The most likely spike train was modeled from the calcium indicator fluorescence changes (Vogelstein et al., 2010; Sadovsky et al., 2011). On this basis cells were assigned a firing onset time for each epileptiform discharge. The number of simultaneous spiking onsets of individual cells was thresholded to define onsets of circuit events (threshold between 0.25% of cells active for populations <400 cells and 0.33% of cells active for populations of 1100 cells). In somatic whole-cell recordings, epileptiform discharges were detected as previously described (MacLean et al., 2005). The overlap of epileptiform discharges was used to determine the coupling reliability (CR) between neocortex and thalamus.

#### Correlating imaging data with electrophysiological data

When computing linear dependencies between imaging traces and electrophysiology traces we down-sampled electrophysiological recordings to match imaging frequency by averaging samples. For a particular thalamic cell, the correlation with the cortical signal had to be significantly larger than 0 (*p* < 0.05, MATLAB) and its absolute value larger than 0.1 to count this cell towards the fraction of correlated thalamic cells (Figures 3C,E).

**Figure 1:**
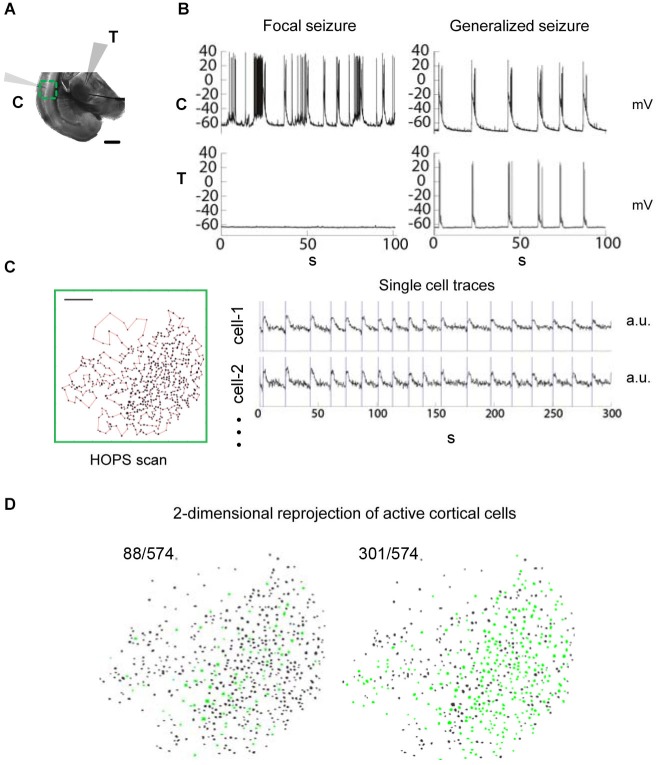
**Observing seizure activity in the thalamocortical (TC) system** ***in vitro* using a combination of multi-photon imaging and electrophysiological recordings in cortex and thalamus**. Functional coupling between brain areas during epileptiform discharges and corresponding local circuit activity are monitored simultaneously. **(A)** TC brain slice incubated in zero-Mg^2+^ bath solution. Recording configuration with electrophysiological patch-clamp recordings in cortical layer L5/6 (“C”) as well as in thalamus (“T”) and multi-photon imaging over cortex (green: field of view). Scale bar 1 mm. **(B)** Dual electrophysiological recordings reveal the coupling status between cortex and thalamus. Left: Focal seizure in cortex. None of the events detected in cortex are seen in thalamus. Right: Generalized seizure with synchronized discharges in thalamus and cortex. **(C)** Multi-photon calcium imaging using Heuristically Optimal Path Scanning (Sadovsky et al., 2011) reveals cortical circuit dynamics with single cell resolution. Scale bar 200 μm. 574 neurons, frame rate 13.64 Hz. Example single cell fluorescence traces with discharge onset detections illustrated by vertical bars. **(D)** 2-dimensional representation of circuit activity. Cells in which activity was detected during cortical discharges are shown in green. The examples represent typical discharges (mean number of active cells) from the recordings shown in **(B)**.

**Figure 2:**
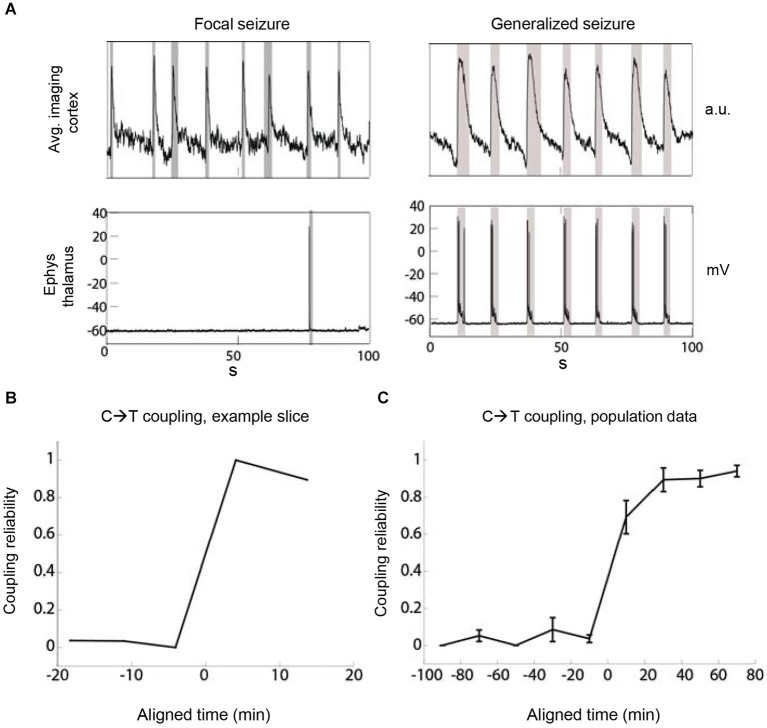
**The recruitment of thalamus by cortex becomes increasingly reliable during epileptiform discharges**. **(A)** The metric “coupling reliability” (CR) quantifies the reliability of thalamic recruitment by cortical discharges as determined over the duration of one recording. Two example recordings show low (left) and high (right) CR in the same slice at different times with values of CR = 1/8 = 0.125 and CR = 7/7 = 1, respectively. Gray boxes indicate detection boundaries of discharges. **(B)** The evolution of CR over time shows a sudden increase of corticothalamic coupling. Example TC slice. The time at which CR crosses 0.5 is aligned at 0. **(C)** Population data. The time at which CR crosses 0.5 was aligned at 0 before averaging. Bin size = 20 min, *n* = 6 slices.

**Figure 3:**
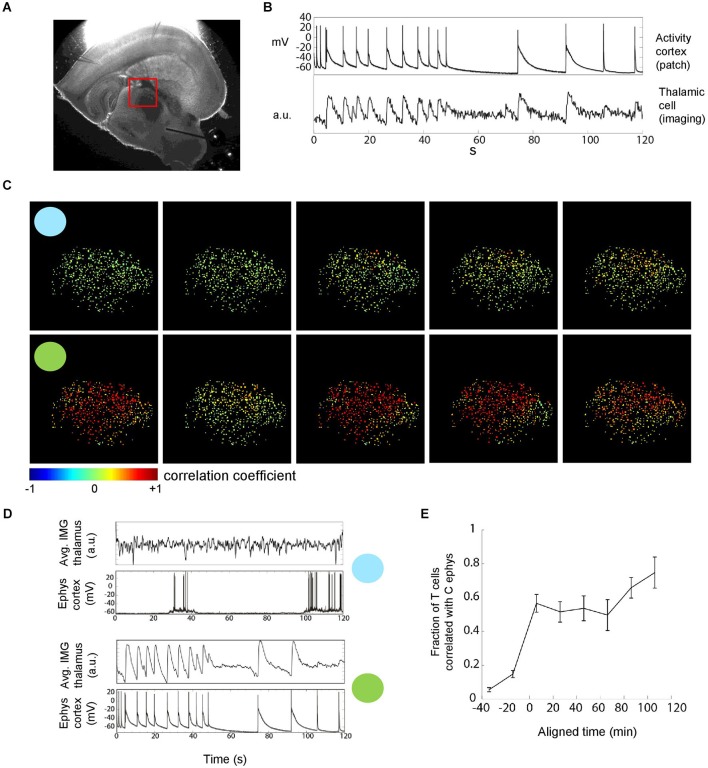
**Thalamic neuronal activity during epileptiform discharges**. **(A)** Thalamic imaging (red box, field of view) and cortical patch-clamp recordings were used to measure the recruitment of individual thalamic cells during epileptiform discharges. **(B)** Cell-wise imaging traces from thalamus were correlated with the (down sampled) cortical electrophysiology trace. The linear correlation coefficient, in this example 0.68, was computed over the whole duration of the recordings (2 min). Cells for which the correlation was significant and larger than 0.1 were counted towards the fraction of cells correlated with cortex as shown in **(E)**. **(C)** Multi-cellular representation of thalamic recruitment. Panels represent cell-wise correlations from consecutive TC recordings. Pseudocolor of thalamic cells encodes the correlation coefficient between their individual activity and cortical activity. Blue and green circles indicate example recordings with focal and generalized seizures, respectively. 454 cells, FOV as shown in **(A)**. **(D)** Imaging average over all cells next to cortical electrophysiology for the two example recordings with circles in **(C)**. Different average discharge intensities reflect the different number of activated cells.** (E)** Temporal evolution of the fraction of correlated thalamic cells. Population data. The time at which the fraction of correlated cells crosses 0.5 was aligned at 0 before averaging. Bin size = 20 min, *n* = 8 slices.

**Figure 4:**
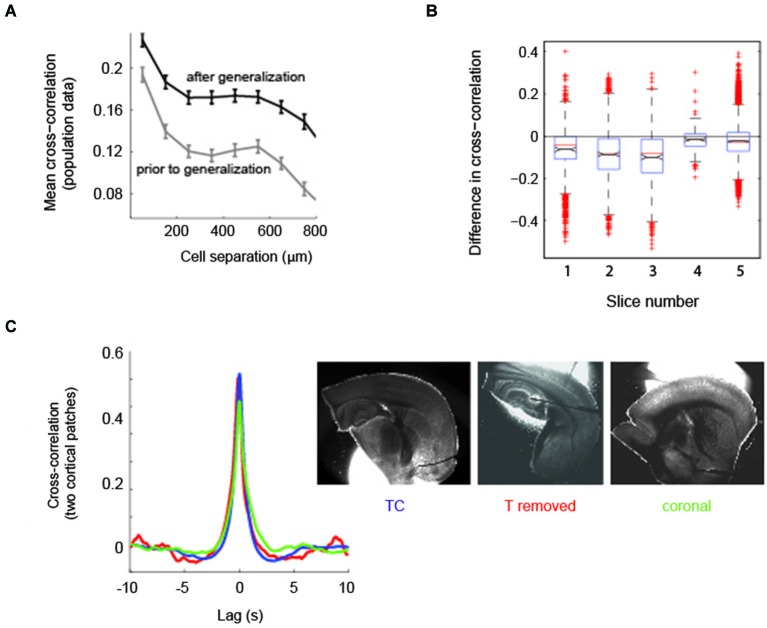
**Aberrant recruitment of thalamus coincides with a state change in cortical activity**. **(A)** Correlations between neurons in cortex are higher after thalamus has been recruited into epileptiform activity. Mean spatial profile of cross-correlation between cortical cells. Gray, before generalization with thalamic recruitment; black, after thalamic recruitment; *n* = 5 slices. **(B)** Distribution of the difference in cell-cell cross-correlations independent of cell-cell distance. Points lower than 0 indicate cross-correlations in cortex were higher after thalamic recruitment. Black lines and notches indicate mean +/− SEM; identical slices as in **(A)**. **(C)** The state change in cortex is independent of thalamus. Left: Averages of pairwise cross-correlations between electrophysiological traces of two randomly patched cells in cortex (mean distance 1278 μm, range 723–2217 μm) show that intra-cortical synchrony does not depend on the presence of thalamus. Blue, TC slices (TC, *n* = 11); red, TC slices from which thalamus was removed (*n* = 4); green, coronal slices (*n* = 3). Right: One example for each slice preparation, out of the slices used for analysis (patch pipets partially removed).

For the analysis of intracortical correlations (Figures 4A,B) we only included slices for which we obtained at least two recordings before spontaneous thalamic recruitment and two recordings after. We also restricted the analysis to cells that were active in at least one event prior to and at least one event after thalamic recruitment. Because changes in dye responsiveness, such as that due to bleaching, could cause changes in correlation across the experiment unrelated to actual changes in activity correlation, we excluded cells that showed non-stationarity in signal strength. To assess the change in signal strength in each cell, we calculated the *z*-scored fluorescence for each event,

(1) $$z\left(t_{i}\right)=\frac{F\left(t_{i}\right)-\langle F_{b}\rangle }{\sigma_{F_{b}}}$$

where 〈*F_b_*〉 and *σ_F__b_* are the mean and standard deviation of the fluorescence over the 2s preceding the fluorescence of event *i* at time *t_i_*, F(t_i_). The *z*-scored fluorescence is a measure of the strength of the signal relative to fluctuations due to noise. Using linear regression, we computed the slope of z(t_i_) and compared it to the distribution of slopes generated by shuffling z(t_i_) in time. If the empirical slope fell outside the 10th–90th percentiles of the shuffled distribution, the cell was discarded from the cross-correlation analysis. Reported correlation values are the linear correlation coefficients of filtered fluorescence traces. For the spatial analysis, we sorted the cell pairs by separation, grouping them in 100-μm bins, and computed the average and standard error of the correlations in each bin. Since one cell may be included in multiple pairs in a given bin, the SEM was calculated using the number of unique cells in the bin, rather than the number of cell pairs, as the number of degrees of freedom.

**Figure 5:**
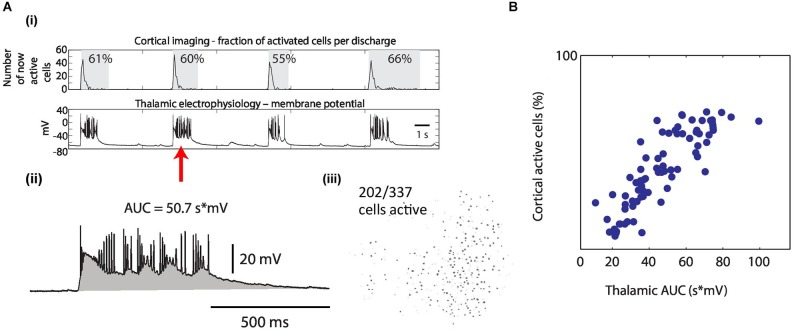
**The intensity of synchronous epileptiform discharges is correlated between thalamus and cortex**. **(A)** Quantification of the intensity of epileptiform discharges. **(i)** Cortical imaging (upper row) simultaneously recorded with thalamic electrophysiology (lower row). Example traces. Quantification of one discharge for both brain areas (red arrow) as follows: **(ii)** In thalamic electrophysiology, intensities were measured as area under the curve (AUC, gray) during the discharge. **(iii)** In cortical imaging, intensities were measured as the percentage of activated cells of ever activated cells (in this example 202/337 = 60%). **(B)** Resulting discharge intensities in cortex plotted against intensities of the same discharges in thalamus, revealing the correlation of epileptiform activity between both brain areas (linear correlation coefficient = 0.85 in this example). Each dot represents one discharge quantification obtained from cortical imaging (all from the same FOV) and from the corresponding, simultaneous thalamic whole-cell recording.

In the quantification of cortical discharge intensities (Figure 5) the fraction of activated cells was defined as the number of cells activated during the course of one epileptiform discharge divided by the number of cells ever active during any recording obtained from the same field of view (FOV). The correlation between cortical and thalamic discharge intensities for a given pair of imaging field-of-view and simultaneous electrophysiological recording had to be significantly different from zero (*p* < 0.05, MATLAB) for this pair to be counted towards correlated fields of view.

Error bars in population data panels represent +/− SEM.

## Results

### Examination of functional coupling between brain areas during epileptiform discharges

To quantify the spread of epileptiform activity in the TC system we employed large-scale multiphoton imaging and electrophysiological whole-cell recordings in the zero-Mg^2+^ model of epilepsy in somatosensory TC slices (Figure 1A). We performed simultaneous patch-clamp recordings in thalamus and cortex to determine the seizure status of the slice, which could either be focal (only one brain area active) or generalized (both brain areas showing synchronous epileptiform activity) (Figure 1B). Simultaneously, we performed rapid imaging of either cortical or thalamic neuronal populations (250–1100 cells) using Heuristically Optimal Path Scanning (Sadovsky et al., 2011). We tracked spiking activity within the imaged neurons loaded with the calcium indicator dye Fura-2AM (Vogelstein et al., 2010; Sadovsky et al., 2011; Figures 1C,D). Our approach allowed us to quantify functional coupling between anatomically distant brain areas (Figure 1B) and the associated local circuit activity (Figure 1D) during epileptiform activity at the same time.

### Localizing seizure onset

First, we localized the site of focal epileptiform activity onset. Specifically, we determined whether cortex or thalamus was more likely to be the site of origin for epileptiform discharges in the TC system, given the same condition of hyperexcitability. Based on inter-areal double electrophysiological recordings in both thalamus and cortex, we determined the anatomical structure where focal seizure onset occurred in every slice. In all cases where we began recordings soon after the application of zero-Mg^2+^, focal activity was first detected in cortex with no activity present in the thalamic patch (*n* = 19 out of 19 slices, compare Figure 1B left panel). None of the preparations showed focal seizure activity initially in thalamus. As a control, we confirmed preserved synaptic connectivity from thalamus to cortex in every slice included in this study by evoking reliable cortical responses via electrical extracellular thalamic stimulation (MacLean et al., 2005). In this manner we ruled out the possibility that the spatial restriction of activity was due to an anatomical disconnect. These results suggest that when both anatomical structures are simultaneously exposed to the same condition of perturbed excitation and inhibition, neocortex is the site of focal seizure onset in the TC system, consistent with previous reports (Coulter and Lee, 1993; Meeren et al., 2005; Gigout et al., 2013).

### Quantification of seizure activity

To gain a more detailed picture of the temporal evolution of the global pathophysiological coupling between thalamus and cortex we quantified the reliability of coupling (CR) between these two brain areas. We defined CR as the fraction of all cortical epileptiform discharges that were simultaneously detected in thalamus (CR = number of discharges detected as synchronous in C and T/number of all discharges detected in C). A CR value of 0 indicated thalamic silence, whereas a CR of 1 meant perfect corticothalamic coupling (Figure 2A). We applied the CR metric to TC recordings comprising cortical imaging and thalamic electrophysiology. In the cases where we initiated imaging prior to the onset of corticothalamic coupling and we also observed eventual strong coupling of thalamus in synchronized discharges (CR < 0.1 in the first available recording and CR > 0.8 in any later recording; *n* = 6 slices), the increase of coupling occurred in a nonlinear fashion (Figures 2B,C). Once coupled simultaneous recordings from a thalamic and a cortical neuron indicated that thalamus almost always discharged synchronously with cortex as 91.5% of thalamic epileptiform discharges coincided with epileptiform discharges recorded in cortex (2575 thalamic discharges, 512 min total recording time, *n* = 25 slices). These data indicated that thalamus is recruited nonlinearly into synchronous discharges with cortex and that the reliability of coupling increases over time.

### Leading role of cortex in individual generalized discharges

Next we studied the spread of epileptiform activity from one brain area to the other at the temporal resolution of individual discharges. We measured the delay between the onset of a given epileptiform discharge in cortex and the onset of the corresponding discharge in thalamus. Previous work has demonstrated that the cortical location for the initiation of epileptiform activity in the zero-Mg^2+^ model is variable (Wong and Prince, 1990). To account for this spatial variability, we evaluated the relative onset timing of epileptiform activity between brain areas using three experimental configurations: (1) imaging in cortex/patch clamp in thalamus; (2) patch clamp -cortex/patch clamp -thalamus; and (3) patch clamp -cortex/imaging -thalamus. Based on patch clamp -cortex/patch clamp -thalamus experiments, the median lag between the detection of an epileptiform discharge in cortex and its detection in thalamus (regardless of subthreshold or suprathreshold depolarization) was 65.9 ms (2199 matched discharges, *n* = 22 slices). The lag between thalamus and cortex was similarly positive when observed in the other recording configurations (imaging-cortex/patch clamp-thalamus: median corticothalamic lag 153.8 ms, 2402 TC discharges, *n* = 35 slices; patch clamp-cortex/imaging-thalamus: median 91.9 ms, 3224 TC discharges, *n* = 27 slices). Across all generalized discharges, cortex was found to lead epileptiform discharges in 82.5% of cases in the imaging -cortex/patch clamp -thalamus configuration (patch clamp -cortex/patch clamp -thalamus: 70.7%; patch clamp -cortex/imaging -thalamus: 67.6%). Thus the larger the sample size of cortical neurons the more likely we first detected activity in cortex consistent with epileptiform discharges in cortex preceding thalamic activity. The sum of evidence indicates that it is likely that generalized discharges originate from aberrant circuit activity in cortex.

### Imaging of thalamus confirms a dominant role for cortex

We next imaged thalamus combined with patch clamp recording of cortical neuron(s) during epileptiform activity to quantify the proportion of thalamic neurons that are recruited into generalized seizures (Figure 3A). We correlated the individual imaging trace of each thalamic cell with the cortical electrophysiological trace (Figure 3B) to determine whether a thalamic cell was participating in a generalized discharge or not. This corticothalamic correlation revealed a nonlinear transition in time from sparse thalamic recruitment to a majority of thalamic neurons being synchronously active during epileptiform discharges (Figures 3C–E). Thus, thalamic imaging showed that the transition from focal epileptiform activity in cortex to generalized corticothalamic discharges not only is reflected by a nonlinear increase in the reliability of synchronous corticothalamic firing (Figure 2) but also a nonlinear increase in the number of thalamic cells recruited into each epileptiform discharge.

### Aberrant recruitment of thalamus coincides with increased pairwise correlation in cortex

Because the progression of cortico-thalamic coupling was nonlinear we analyzed cortical circuit activity before and after thalamic recruitment to determine whether a change in cortical circuit activity was indicative of thalamic recruitment into epileptiform discharges. Specifically we computed pairwise correlations in activity across the population of imaged cortical cells before and after generalization defined as recruitment of thalamus. We restricted the analysis to experiments in which we had at least two recordings in each condition (*n* = 5 slices). Recordings contained an average of 46 ± 27 discharges prior to thalamic recruitment and an average of 63 ± 37 discharges after thalamic recruitment. We found that, on average, cortical correlations fell off with the intersomatic distance between cells, both before and after thalamic recruitment (Figure 4A; *n* = 5 slices). However, cortical correlations were significantly higher regardless of intersomatic distances after the recruitment of thalamus (Figure 4B; *p* < 1e-5; 518 cells across the same 5 slices shown in Figure 4A). This data indicates that increased coherence of cortical neuronal activity is coincident with aberrant global recruitment of thalamus.

### Changes in neocortical activity are independent of thalamus

To confirm that the changing level of pairwise correlations found in cortex was independent of thalamus we compared the cross-correlation between two simultaneously patch clamped cortical neurons in slices with and without TC connectivity. The latter group was comprised of TC slices in which we physically removed thalamus (*n* = 4, patch distances in cortex 1381 +/− 170 μm) and coronal slices (*n* = 3, patch distances in cortex 1098 +/− 165 μm SEM), which do not contain intact TC connectivity (Figure 4C, right panel). Comparison of double-patch cross-correlations showed that the correlated cortical state in TC slices and both types of slices without thalamic influence showed no qualitative difference (Figure 4C, left panel). For statistical analysis, both types of slices without TC connectivity (coronal and thalamus removed) were pooled (*n* = 7) and compared against TC slices (*n* = 11, patch distances in cortex 1290 +/− 144 μm SEM). Correlations at zero lag were statistically indistinguishable between slices with and without corticothalamic connections (*p* = 0.285, Wilcoxon rank sum test; mean correlation coefficient for two cortical patches in TC slices = 0.61 +/− 0.05 SEM, mean correlation coefficient for slices without connectivity = 0.56 +/− 0.08 SEM [subdivided: coronal 0.52 +/− 0.19, thalamus removed 0.59 +/− 0.02 SEM]), indicating that cortical synchrony during epileptiform discharges can be established without the influence of thalamus. We conclude that the level of pairwise correlation in cortex that is capable of recruiting thalamus into generalized discharges occurs in cortex regardless of the presence of an intact corticothalamic/thalamocortical loop.

### Graded coupling between thalamus and cortex

Finally, we set out to evaluate whether the intensity of epileptiform activity in cortex and thalamus was correlated following the recruitment of thalamus into joint discharges. We considered the following hypothesis. Activity in cortex acts as a trigger of activity in thalamus with the intensity of the resulting thalamic activity exclusively determined by local thalamic factors. The alternative hypothesis was that discharge intensity in thalamus tracks discharge intensity in cortex suggesting a dependency of thalamic intensities on cortical activity even after the entrainment of both structures into generalized seizures. To evaluate these two possibilities we determined the amount of correlation of the intensity in both brain areas during synchronous epileptiform discharges (Figures 5A,B). Discharge intensity in cortex was quantified by the fraction of activated cells within an imaging FOV. Discharge intensity of the same epileptiform discharge in thalamus was quantified by the integral of depolarization recorded in a patch clamped thalamic neuron (Figures 5A,B). We found a significant correlation between the intensity of thalamic and cortical activity in 18 out of 21 datasets (median linear correlation coefficient for the 18 significant correlations = 0.70, range 0.30–0.88). This data suggests that even in the pathophysiological condition of generalized epileptic seizures thalamus and cortex act as a coupled system with correlated intensities of activity.

## Discussion

### Cortex dominates the initiation of epileptiform discharges

We first determined how often focal epileptiform activity arises in thalamus and in cortex in the acute TC brain slice preparation in combination with zero-Mg^2+^ induction of seizures. Consistent with previous reports (Coulter and Lee, 1993), we found that when thalamus and cortex are both exposed to the same condition of perturbed excitation and inhibition, cortical circuits are the dominant source of epileptiform drive in the TC system. In all cases in which we were able to record the initial aberrant activity, cortex was the primary focus of epileptiform discharges while thalamus was still silent. After recruitment of thalamus into epileptiform activity, the majority of individual epileptiform discharges arose in cortex and spread to thalamus. We speculate that the true fraction of individual epileptic discharges initiated by cortex lies much closer to 100% than the 68–83% we measured due to cortical undersampling. Despite our large imaging field of view, the aperture was not large enough to cover the full extent of the cortex in our preparation. Epileptiform activity can begin in changing, random locations in cortex (Wong and Prince, 1990), hence it is likely that we missed the true cortical initiation site of some of the epileptiform discharges. However, we cannot exclude the possibility that in a minority of cases reverse lags reflect the propagation of genuine thalamic discharges to cortex. We note that we confirmed that each slice contained intact synaptic connectivity from thalamus to cortex using extracellular stimulating in thalamus and recording the cortical response. The long average delay of ~66 ms between activity onset in a randomly patched L5 cell in cortex and a randomly patched TC cell was surprising. However our data suggest that the intracortical build-up of coherence is the basis of a population-wide recruitment of thalamus, and we speculate that this might happen on this time scale. Possibly metabotropic receptors are also involved as they have shown to operate on exactly this time scale at room temperature *in vitro* (McCormick and von Krosigk, 1992; Turner and Salt, 1999).

Our data indicating a dominant role for cortex in seizure generation in the TC system is consistent with previous reports that employed a variety of epilepsy studies, including ionic, pharmacological, and genetic models, examined both *in vitro* (Coulter and Lee, 1993; Gigout et al., 2013) and *in vivo* (Steriade and Contreras, 1998; Meeren et al., 2002; Coenen and Van Luijtelaar, 2003; Meeren et al., 2005). The consistency of this result in many different model systems strongly suggests that neocortex has an intrinsic predisposition for seizure generation. In the case of the zero-Mg^2+^ model, there is no obvious reason, other than recurrent connectivity, for the iniation of aberrant discharges to be biased toward being more likely in cortex. Under zero magnesium, two effects act together to cause epileptiform activity, namely enhanced excitation due to the removal of the magnesium block at NMDA receptors (Walther et al., 1986; Mody et al., 1987; Traub et al., 1994) and largely reduced inhibition (Whittington et al., 1995; Trevelyan et al., 2007). A site-specific difference in cortex as compared to thalamus for one or both of these effects might translate into cortex being the site for focally initiating hyperexcitability. However, there appears to be no such bias. First, TC neurons express NMDA receptors (Salt, 1986; Miyata and Imoto, 2006; Hsu et al., 2010). Because we bath applied zero-Mg^2+^, unblocking of NMDA receptors can be expected to occur in both cortex and thalamus. We note that the relative density of receptor-channels, to our knowledge, has not been quantified. Second, inhibition, which is reduced in this model of epilepsy, is equally present in thalamus and cortex. The main source of inhibition at TC cells is afferents from the reticular thalamic nucleus and reduced inhibitory drive onto thalamus can, despite the reduction of rebound bursts, induce thalamic hyperexcitation (Lacey et al., 2012). We also note that NMDA receptors are present on the neurons of the reticular thalamic nucleus as well (Lacey et al., 2012), which under zero-Mg^2+^ might cause the opposite effect, an enhanced inhibition of TC cells, but at the same time, as a consequence of this, possibly also an increase in rebound activity. In the electrophysiological recordings presented in this study, however, none of the epileptform discharges (thousands of events) in TC cells arose from a rebound event following hyperpolarization (cf. Figures 1B, 2A). Yet, the combined effect of fading inhibition, NMDA-R unblocking on TC neurons, and NMDA-R unblocking on reticular neurons on the net excitability of thalamo-cortical cells remains unknown. We conclude that cortex has a predisposition for seizure generation under zero-Mg^2+^ as well as in other types of epilepsy models. Hence, the most consistent explanation, as previously postulated, is the prevalence of local recurrent synaptic connections in cortex (McCormick and Contreras, 2001).

### Short-range circuit activity in cortex changes when thalamus is recruited

The appearance of an epileptic focus alone is not sufficient for the transmission of aberrant activity into secondary areas. Rather our data suggest that a certain threshold of local discharge intensity needs to be crossed to recruit distant sites. Making use of the single cell resolution of our imaging approach, we determined that the pairwise correlation within large populations of neocortical cells increases at the time when thalamus is recruited into time-locked aberrant discharges of both brain areas. Importantly, this change in the local circuit activity in cortex also occurred when thalamus was not present in the slice preparation (Figure 4C), indicating that reciprocal reinforcement of activity between the primary and the secondary brain area is not necessary for the local change in circuit correlational structure. This finding is in line with the clinical notion of focal seizures which might not reach the level of coherence which is required to recruit larger areas of the brain.

### Discharge intensity in thalamus is correlated with discharge intensity in cortex during generalized seizures

By quantifying the intensity of simultaneous discharges in cortex and thalamus we were able to show a link between cortical and thalamic discharge intensities. After thalamus and cortex are recruited into joint synchronous discharges in the generalized state, we found that both structures act as a closely coupled system typified by correlated discharge amplitudes. These data suggest that cortex as the initial focus not only determines the time of discharge onset in the anatomically distant brain area of thalamus but also controls the intensity of thalamic discharges as indicated by correlated discharge amplitudes in combination with a temporal lead of cortex. The fact that the coupling between two brain structures can remain graded and dynamic in the condition of generalized seizures might inform future models which aim at improving the local brain stimulation protocols used for the treatment of drug-resistant epilepsies (Fisher, 2011).

## Conflict of interest statement

The authors declare that the research was conducted in the absence of any commercial or financial relationships that could be construed as a potential conflict of interest.
